# Immunogenic Cell Death Role in Urothelial Cancer Therapy

**DOI:** 10.3390/curroncol29090526

**Published:** 2022-09-18

**Authors:** Reza Yadollahvandmiandoab, Mehrsa Jalalizadeh, Keini Buosi, Herney Andrés Garcia-Perdomo, Leonardo Oliveira Reis

**Affiliations:** 1 UroScience, School of Medical Sciences, University of Campinas, UNICAMP, Campinas, Sao Paulo 13083-970, Brazil; 2 Division of Urology/Urooncology, Department of Surgery, School of Medicine, Universidad del Valle, Cali 72824, Colombia; 3 Center for Life Sciences, Pontifical Catholic University of Campinas, PUC-Campinas, Sao Paulo 13087-571, Brazil

**Keywords:** bladder cancer, immunogenic cell death, immunotherapy, immune checkpoint inhibitors, scoping review

## Abstract

Purpose: Bladder cancer is the 13th most common cause of cancer death with the highest lifetime cost for treatment of all cancers. This scoping review clarifies the available evidence on the role of a novel therapeutic approach called immunogenic cell death (ICD) in urothelial cancer of the bladder. Methods: In accordance with the recommendations of the Joanna Briggs Institute, we searched MEDLINE (Ovid), EMBASE, CENTRAL databases, and supplemented with manual searches through the conferences, Google scholar, and clinicaltrials.gov for published studies up to April 2022. We included literature that studied molecular mechanisms of ICD and the role of certain danger-associated molecular patterns (DAMPs) in generating ICD, safety and efficacy of different ICD inducers, and their contributions in combination with other urothelial cancer treatments. Results: Oncolytic viruses, radiotherapy, certain chemo/chemo radiation therapy combinations, photodynamic therapy, and novel agents were studied as ICD-inducing treatment modalities in the included studies. ICD was observed in vitro (murine or human urothelial carcinoma) in ten studies, eight studies were performed on mouse models (orthotopic or subcutaneous), and five clinical trials assessed patient response to ICD inducing agents. The most common studied DAMPs were Calreticulin, HMGB1, ATP, and Heat Shock Proteins (HSP) 70 and 90, which were either expressed on the cancer cells or released. Conclusion: ICD inducers were able to generate lasting antitumor immune responses with memory formation in animal studies (vaccination effect). In clinical trials these agents generally had low side effects, except for one trial, and could be used alone or in combination with other cancer treatment strategies in urothelial cancer patients.

## 1. Introduction

According to estimates, about one million cells die every second in the human body because of normal tissue turnover, and throughout this process the immune system is frequently exposed to dead cells, as well as during damage and infection [1,2]. Physiological mechanisms must be able to distinguish between various types of cell death to effectively eradicate pathogens, promote healing, and prevent autoimmunity. The immune system decides whether the cell death is immunogenic or tolerogenic.

Tolerogenic cell death occurs in the absence of pathogens and does not cause any immune response. Conversely, immunogenic cell death (ICD) is defined as a kind of cell death that triggers an immune response to dead-cell antigens, especially when those antigens come from cancer cells [2]. According to the ICD concept, many kinds of anti-cancer treatments, such as gamma-irradiation, chemotherapeutics, immunotherapy, and photodynamic therapy [3,4,5] provoke immunogenic cell death, most commonly apoptotic cell death. These apoptotic cells present certain danger-associated molecular patterns (DAMPs), including translocation of calreticulin (CRT) to the plasma membrane, secretion of adenosine triphosphate (ATP) from the cytosol into the extracellular space, translocation of HSP70/HSP90 to the cell surface, and release of high mobility group box 1 (HMGB1) from nucleus into the extracellular space [6,7]. These DAMPs are considered as essential hallmarks for cell death to be considered as ICD, and the absence of any one of them reduces the immunogenicity in cell death [8,9,10]. DAMPs mainly cause dendritic cells (DCs) to be attracted to the tumor bed, where they engulf tumor cells. Then, mature DCs present antigens to tumor-specific cytotoxic T lymphocytes (CTLs), which ultimately results in CTLs killing the tumor cells [10].

Bladder cancer (BC) represents over 90% of all urothelial cancers, and with about 550,000 recognized new cases per year, is one of the top 10 most prevalent cancer in the world. It accounts for about three percent of all new cancer diagnoses and, with more than 200,000 deaths per year, is the 13th main reason for cancer death [11,12]. Men have a higher (9.6 per 100,000) global age-standardized incidence rate (ASR) than women (2.4 per 100,000). The prevalence of bladder cancer differs greatly by region. In the US and Europe, the incidence of BC is approximately 15 cases per 100,000 people per year [12,13]. Male sex, tobacco smoking, chemical exposure and family history are the main risk factors for this cancer [13]. The 5-year overall survival (OS) rate for BC is about 77%. Although, after five years following diagnosis, just 6% of individuals with metastatic BC are still alive. [14]. Due to the long-term survival rate and the use of intensive surveillance, BC has the highest lifetime cost for each patient of all cancer types [15].

Muscle-invasive bladder cancer (MIBC) and non-muscle invasive bladder cancer (NMIBC) are different types of bladder cancer [11]. NMIBC accounts for 80% of bladder cancer diagnoses and is found to carry mutations in the DNA helicase ERCC2, tumor suppressor TP53, and fibroblast growth factor receptor 3 (FGFR3) [16]. MIBC comprises about 20% of bladder cancer cases and is known to have mutations in tumor suppressor TP53, FGFR3, transcriptional activator ELF3, histone demethylase KDM6A, tumor suppressor RB1, and DNA helicase ERCC2 [11]. NMIBC is typically detected locally in the urothelium (stage Ta) or lamina propria (stage T1), whereas MIBC is more advanced with its invasion to the muscle (stage T2) or beyond (stages T3 and T4) [11,16]. In addition to surgery, patients with BC may receive radiotherapy, chemotherapy such as Mitomycin C, Adriamycin, Epirubicin, Platinum-based agents, Gemcitabine, and Doxorubicin or Bacillus Calmette–Guerin (BCG) immunotherapy. However, these treatment results remain poor [17,18]. In recent years, five immune checkpoint inhibitors (ICIs) including atezolizumab, pembrolizumab, durvalumab, nivolumab, and avelumab, have been approved by the European Medicines Agency (EMA) and US Food and Drug Administration (FDA) to treat metastatic and advanced bladder cancer, and have shown striking results [19].

Over the past few decades, major progress has been made in understanding the cellular and molecular mechanisms of different types of cell death. These advances have shown molecular disturbances in these pathways in different diseases, which could be potential targets for more effective therapy. Given that cancer therapy is based on the induction of cell death in cancer cells, knowledge about cell cycle regulation has led to several opportunities for novel cancer therapy [20,21]. Considering the important role of immunogenic cell death (ICD) in cancer therapy, we aimed to better understand the ICD role in urothelial cancer therapy.

## 2. Materials and Methods

We conducted a scoping review. A scoping review is an approach to systematically prospect the scope and size of a body of literature on a subject [22,23], which allows more exploration by including unlimited study designs, settings and outcomes. The design and implementation were guided by a methodological framework developed by Arksey and O’Malley [22], refined by Colquhoun et al. [24], and described by the Joanna Briggs Institute [25]. The PRISMA-model [26] was used to organize the information, and the recommendations described in PRISMA-ScR were followed [27].

### 2.1. Inclusion and Exclusion Criteria

This review focused on evaluating the relationship between ICD and its disturbances in urothelial cancer therapy. Inclusion and exclusion criteria were the following: (a) studies carried in vitro or in vivo that include theoretical information regarding the role of immunogenic cell death in urothelial cancer therapy; (b) no limitation in the published language. We excluded observational studies, reviews, letters, editorials, conference abstracts, articles with unrelated topics, replies from authors, articles without full texts access.

### 2.2. Information Sources

A comprehensive search of the literature published until April 2022 was conducted following Emtree language, medical subject headings (MeSh), and related text words, using the databases Embase, MEDLINE (Ovid), and the Cochrane Central Register of Controlled Trials (CENTRAL). This search was supplemented with manual searches of the reference lists of extracted articles and relevant articles identified through the conferences, Google scholar, and clinicaltrials.gov, to ensure that no relevant reference was missed. For detailed search strategies see Appendix A.

### 2.3. Data Extraction

Titles and abstracts were screened separately by the first two authors with a portion of articles double screened by both for application of pre-specified inclusion and exclusion criteria. Data were extracted by the first author and assessed by the second author. Extraction was guided by a standardized form for this review and approved by the authorship team and included the following information from each article: study reference, type of cancer or cell lines, sample size, treatment, measured DAMPs, and immune response.

## 3. Results

**Figure 1:**
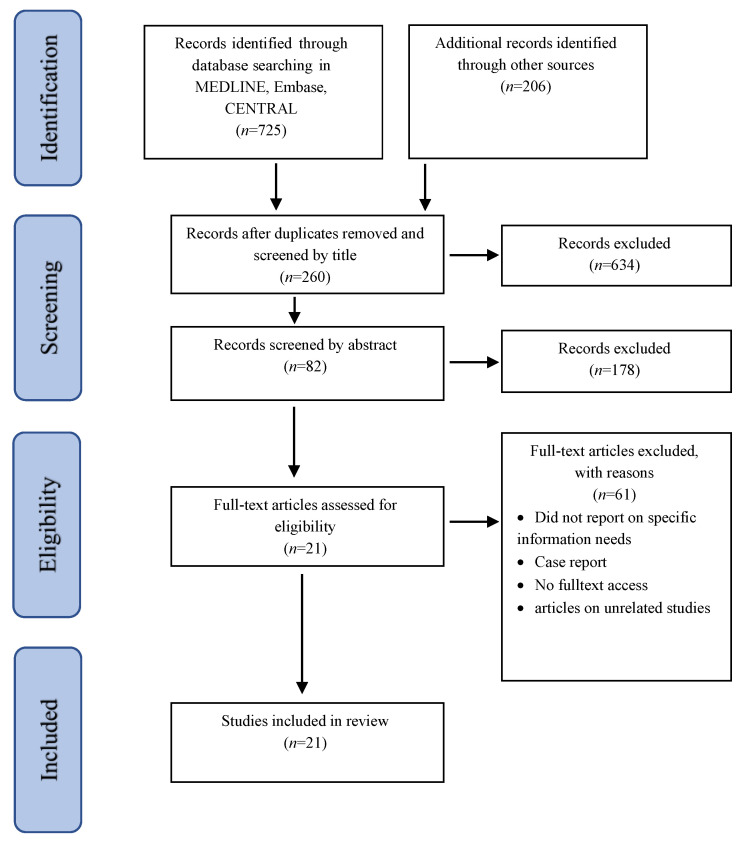
Process of identification and inclusion of studies. PRISMA Flow diagram.

The primary search yielded a total of 931 citations (725 from electronic databases and 206 from other sources). After removing the duplicates, the first author independently screened the remaining 894 titles and extracted those clearly not meeting the inclusion criteria. Abstracts of potentially eligible studies (*n* = 260) were then reviewed independently by the first and second authors. Full-text of the remaining studies (*n* = 82) were obtained and screened against the inclusion criteria. Any disagreements regarding inclusion were resolved by discussion. The remaining 21 studies met the inclusion and exclusion criteria and are included in this review (see Figure 1 for the PRISMA flow diagram).

Table 1 shows main ICD inducers based on their immunogenicity and DAMP response-mediated and Table 2. the clinical trials involving ICD inducing agents.

## 4. Outcome

### 4.1. Oncolytic Viruses

Viruses are known to cause cell death while creating an immune response against the infected cell; this response is by definition an ICD [49]. Different viruses have been studied in recent years as “oncolytic viruses” due to their ICD potential. Some of these viruses naturally induce ICD and some were genetically modified (recombinant form) to enhance their ICD potential or make them target cancerous cells specifically or enhance their immune stimulation potential. Oseledchyk et al. [43] investigated the Newcastle disease Virus (NDV) in its original form because this paramyxovirus has a natural tendency towards human cancer cells. They observed increased immune cell infiltration plus improved response to Immune Checkpoint Inhibitors (ICI). The virus caused apoptosis and its ICD effect was independent of lysis.

Liljenfeldt et al. [37] used a recombinant form of adenovirus that induced the expression of CD40 ligand on its host cells (called RAd-CD40L). The virus in combination with chemotherapeutic agent 5-fluorouracil induced enhanced systemic immunity, tumor shrinkage and survival in mice. The same recombinant adenovirus (called AdCD40L) was tested in a phase I/IIa clinical trial on eight patients with bladder cancer [48]. Post-treatment biopsy of those patients showed T cell infiltration, increased IFN-γ marker and reduced load of malignant cells; however, they did not have any controls. Circulatory T regulatory cells were also reduced in all of their patients.

Pseudovirion: Hojeij et al. [35] produced a modified human papillomavirus (HPV) that did not have the ability to replicate; hence the term pseudovirion. The pseudovirion was used to transfer a “suicide gene” into cancer cells. The suicide gene was herpes simplex virus thymidine kinase due to its ability to turn ganciclovir into its toxic form. Ganciclovir is a medication that is activated only in cells that contain thymidine kinase and causes cell death [50]. Hojeij et al. [35] observed that this combination induced ICD in vitro, reduced in vivo tumor growth and increased mice survival.

Lichtenegger et al. [34] studied a recombinant adenovirus that specifically replicates in cancer cells because these cells have nuclear localization of YB-1. YB-1 is a human oncogenic transcription factor and in this study was shown to be highly expressed in multiple bladder cancer cell lines. They showed ICD induced by this recombinant virus through release of HMGB1 and HSP70 in higher levels than the wild type virus and the control. The virus had a higher ICD induction capacity than doxorubicin, which is a known ICD-inducing chemotherapeutic drug.

Vesicular stomatitis virus containing both human and mouse GM-CSF were both tested in vitro by Rangsitratkul et al. [46]. In vitro they were able to induce ICD by release of HMGB1 and ATP plus expression of calreticulin. In vivo, the virus enhanced immune cell activation, tumor immune cell infiltration, and improved survival.

Annels et al. [29] studied Coxsackievirus A21 with the natural ability to target intracellular adhesion molecule-1 (ICAM-1). They discovered that the virus must be alive for its in vitro oncolytic effect and had varying oncolytic potency in different bladder cancer cell lines. The variation in oncolytic potency was associated with inability of the virus to enter the cytoplasm of some of the resistant cell lines. Some resistant cell lines also had low surface ICAM-1 expression. Mitomycin C treatment of the cells increased ICAM-1 expression only in those cell lines that were already capable of expressing ICAM-1. Mitomycin C also increased the oncolytic effect of Coxsackievirus A21 by the induction of apoptosis. The virus alone enhanced ICD markers by ecto-calreticulin expression and HMGB1 release. In their mouse model, injecting the combination of tumor-virus lysate caused an anti-cancer vaccination effect. However, the tumor cell line that was injected in the mouse was already modified to increase ICAM-1 production. In short, they saw oncolytic ability of the virus only in tumors that produce ICAM-1, using living virus, and MMC only enhanced oncolytic ability of the virus on cell lines that were already susceptible. In their mouse model, only CD4+ cells were shown to modulate AVE, as mice depleted with CD4+ were unable to respond with AVE while CD8+ and NK-depleted mice had intact responses. The same authors later tested this virus in a phase I clinical trial on patients with NMIBC [45]. They intravesically introduced the virus to 16 patients before their scheduled TURs; some patients also received low dose MMC in one of the instillations to enhance the oncolytic effect of the virus. Study of the resected tumors showed that the virus selectively replicated in the tumor cells. The patients’ urinary HMGB1 showed escalating levels following treatment, indicating the potential of this therapy to induce ICD only in cancerous cells. Up-regulation of immune checkpoint inhibitors PD-L1 and LAG3 and increased numerous cytokine production were also observed.

### 4.2. Anticancer Vaccination Effect

If an agent generates immunogenicity against cancer, its indirect anti-cancer effects are expected to remain in the immune system’s memory. Injection of such agents inside the tumor in mice has been shown to cause anti-tumor activity that extends beyond the injection site; i.e., distant tumors or tumors that are injected later are affected as well. Rejection of tumor cells in animal models after their immune system is exposed to cancerous cell compounds is called the anti-cancer vaccination effect (AVE).

Oseledchyk et al. [43] created two subcutaneous tumors in each mouse and injected their therapy agent (oncolytic virus NDV) locally inside only one of the tumors. They observed immune cell infiltration in the uninjected tumor as well as the injected tumor. This distant response could be due to traveling activated immune cells. A shift of inhibitory T cells to the effector phenotype was observed in their study, which is usually due to DC maturation [51]. The distant response can also be because their therapeutic agent is a replicating virus that can spread in the body and infect both tumors.

Garg et al. [38] investigated the role of ICD in generating AVE by studying rat cancer cell lines that are naturally resistant to AVE (AY27), and compared it to the murine cell line CT26 without this resistance. First, they induced apoptosis using the ICD-inducing agents Hypericin-photodynamic (Hyp-PDT) or mitoxantrone (MTX), then they injected the dying cells to one flank of the mouse or rat. The same animal was then challenged with live tumor cell on the other flank. They observed no AVE in rats injected with the AY27 cell line. They further showed that this was due to defective ICD in this cell line from failure to expose CRT on their surface.

Oresta et al. [41] observed AVE from high-dose-short-exposure of MMC. BC cells treated with a high dose of this chemotherapeutic agent for one hour underwent apoptosis enough to stimulate a lasting immune response in the mice that received them, and subsequently generated AVE. Previous studies that used low doses of the drug failed to show its ICD activity in short exposure times. Their study concluded that ICD generated from MMC relied on a cascade of events originating in cancer cell metabolism: oxidative phosphorylation causing increased mitochondrial permeability, followed by cytoplasmic release of mitochondrial DNA, and subsequent activation of inflammasome leading to IL-1 β secretion and eventually maturing dendritic cells.

### 4.3. Photodynamic Therapy (PDT)

PDT is a combination of three agents: photosensitizer, light, and oxygen. Together these three agents increase reactive oxygen species, causing cell apoptosis or necrosis [52]. Gerg et al. [42] used hypericin as their photosensitizer, an agent that disrupts normal endoplasmic reactions if activated by light. The study showed ICD induction through reactive oxygen species (ROS) production in the endoplasmic reticulum (ER stress) of human and mice bladder cancer cell lines. ICD in this study was shown by expression of surface calreticulin and active ATP release before apoptosis, subsequently leading to DC maturation and activation. The same authors later showed that autophagy regulates this response and reduces ICD (contrary to the Xu et al. [36] study). Attenuating autophagy in the second study enhanced immunogenicity of the cells; the authors postulated that autophagy protects cancer cells from ICD [44].

### 4.4. Inhibitory DAMPs (iDAMPs)

This type of damage-associated molecular pattern is shown to reverse the effect of DAMPs by reducing the immunogenicity of the cell death [53]. Nikolos et al. [40] showed that inhibition of prostaglandin-E2, a known iDAMP, increases ICD and turns chemo-immunotherapy unresponsive tumors into T-cell inflamed responsive tumors. The same authors previously showed concomitant release of iDAMP and DAMP as a mechanism of ICD resistance and failure of AVE generation when gemcitabine was added to chemotherapy regimen [39].

### 4.5. Radiotherapy, Chemotherapy and Combination Therapy

Radiotherapy is a known inducer of ICD [54]. Zeng et al. [30] showed that radiating the human bladder cancer cell line BT-B increases apoptosis and cell surface expression of calreticulin, HMGB1 and HSP70. The supernatant of irradiated bladder cell culture was capable of maturing a DC culture.

Combination of chemo and radiotherapy on top of checkpoint inhibitor therapy was shown to enhance patient response to treatment by Fukushima et al. [31]. They further tested this theory on mouse models and observed better survival of the mice with orthotropic bladder cancers if treated with all three therapies. They also showed that this was due to enhanced ICD.

In a clinical trial, Galsky et al. [47] attempted to enhance survival of metastatic urothelial cancer patient by combining gemcitabine, cisplatin, and ICI agent ipilimumab, through enhancing ICD generation in the treatment. The trial showed a high risk of grade 3 and above adverse events (81%) and the median overall survival of the patients was 13.9 months. The patients’ serum level of HMGB1 did not increase following this treatment. Some patients had increased CD4+ in their circulation, which correlated with better survival.

### 4.6. New Therapeutic Agents

Norcantharidin is a demethylated analog of cantharidin and has been used to treat cancer in China since 1984. Xu et al. [36] tested this drug’s ability to induce ICD in an acidic environment because some medications lose their potency at low pH. They concluded that this drug increased surface calreticulin and is capable of inducing DC maturation even in an acidic environment. The drug was also able to increase T cell infiltration of mouse tumors and prolong their survival. Unlike the study by Gerg et al. [42], this study showed that autophagy mediated ICD by showing that inhibiting autophagy blocked calreticulin exposure and DC maturation.

Docosahexaenoic acid (DHA) is a dietary polyunsaturated fatty acid with anticancer potential. Molinari et al. [33] observed surface exposure of calreticulin on bladder cancer cell lines after treating the cells with DHA.

Capsaicin, the spicy component of chili pepper, is a known apoptosis inducer in cancerous cells [55]. D’Eliseo et al. [32] showed this molecule’s ability to induce ICD in apoptotic human bladder cancer cell lines by increasing surface calreticulin, HSP90, and HSP70 plus ATP release. The same authors [28] later showed that capsaicin induced apoptosis and maturation of neighbor ingDCs. DC maturation was blocked by silencing CD91, which is considered a DAMP receptor.

## 5. Discussion

In the last few years, immunogenic cell death (ICD) has been recognized as a novel and important pharmacological strategy to improve the effectiveness of cancer treatment and relieve the suffering of cancer patients [56,57]. ICD inducers, such as chemotherapy, oncolytic virus therapy, radiotherapy, and photodynamic therapy by stimulating ICD can coordinate the communication between dying cancer cells and immune cells to sequentially trigger antitumor innate and adaptive immunity [56,58,59]. An ideal ICD inducer should be an efficient activator of necrotic or apoptotic cell death, induce the release of multiple DAMPs, induce ER stress and ROS production, target both primary tumor and metastasized cells, have negligible inhibitory effects on immune cells, escape drug-efflux pathways, and counteract immunosuppressive responses [60,61]. Once an ideal ICD is discovered for bladder cancer, it can hopefully replace radical cystectomy for frail elderly patients who have a high chance of severe surgical complications [62].

In vitro and in vivo studies included in this review all show promising results of ICD-inducing agents on urothelial cancer. ICD-inducing agents were also capable of inducing an anti-tumor vaccination effect, which is an advantage of these agents in preventing recurrence and metastasis. However, Bacillus Calmette-Guerin (BCG), the mainstay of treatment for NMIBC, also showed “promising results” in vitro and in vivo, while in practice proved to only increase survival of patients by 9.2% [63]. As a result, we suggest future in vitro and animal studies should all have a BCG group as the control in addition to neutral vector control. Comparing to BCG will be more informative, as BCG has been extensively studied in human patients and has been routinely prescribed in practice. Laboratory comparison of a new treatment modality to BCG will, therefore, be more helpful in predicting the clinical response in actual patients. Chemotherapeutic agents that are well studied on human patients in various peer-reviewed trials are also good controls, and comparing the new treatment to them will be more informative than comparing new treatments to neutral controls.

Using new treatments in human patients can be more challenging than a control-environment laboratory study. The clinical trials by Annels et al. [45] is a good example. While administering the virus to their study patients, they observed multiple catheter-induced infections, which responded to antibiotics in a relatively large proportion of their patients (6/15). The result of their study may, therefore, be attributed to both the virus and the bacterial infection that was unintentionally introduced.

As we have learned from the COVID-19 pandemic, a mutated virus can spread rapidly in healthcare facilities. We therefore encourage caution in research that studies these new therapeutic agents, especially those viruses that can be airborne (e.g., coxsackievirus is one of causes of common cold). The difference between oncolytic viruses, BCG and other treatment modalities is that a virus replicates very quickly, and has the ability to spread from one human to another. We therefore suggest laboratories to develop tests to identify dangerous mutations. We also strongly suggest clinical trials to isolate patients receiving these treatments (especially their bathroom) until PCR testing shows the patient is no longer shedding the virus. The difference between oncolytic viruses and attenuated virus vaccines is that oncolytic viruses are designed to maintain lethality and are prescribed in high doses. Mass replication of these viruses always has the possibility of creating de novo mutations.

Animals in immunological studies are used due to their intact immune system. However, we would like to remind readers that most mice and rats are kept in sterile environments their entire life. Their immune system is therefore intact but has usually not been exposed to any pathogen. We therefore encourage researchers to design studies while considering the immaturity of the immune system of laboratory grown animals.

Lastly, antibodies have different affinities at different pH levels and temperatures [64,65]. This can affect any immunological study that uses antibodies, including flow-cytometry and simple staining. We encourage researchers to control pH and temperature during all steps of their procedures (including extraction processes) and report them in the study to increase reproducibility of their results. For example, extracting peripheral mononuclear cells on ice may isolate a different set of cells than extracting them in room temperature. We encourage researchers to report these nuanced details.

## 6. Conclusions

Measuring the ICD ability of new agents or new combination therapies can be a reliable prediction tool in assessing bladder cancer responses to therapy in clinics. However, all current studies are in pre-clinical and early-stage clinical trials with limited sample size and controls. We encourage researchers to diligently report every nuance of their procedures to improve reproducibility of their results as well as including a gold standard treatment as a control (e.g., BCG). We also strongly suggest stringent protocols for isolation of patients who are receiving live replicating virus therapy.

## Figures and Tables

**Table 1:** Overview of main ICD inducers based on their immunogenicity and DAMP response-mediated. * Vaccination effect: Rejection of tumor cells in animal models after their immune system is exposed to cancerous cell compounds is called anti-cancer vaccination effect.

ICD Inducer	Tumor Type	Type of Cell Death	Sample Size	DAMPs	Other Outcome	Immune Response	References
Capsaicin	T24 and SD48 human BC cell line	Apoptosis	-	-	CD91 acted as DAMP (CRT and HSP90/70) receptor	DC activation shown by CD86 and CD83 upregulation	[28]
Coxsackievirus A21 (CVA21)	Multiple human BC cell lines and Orthotopic murine model	Apoptosis	-	CRT HMGB1 IFN	-	Vaccination effect * observed after injection with MB49 undergoing ICD from virus	[29]
Radiotherapy	Human BC cells BT-B	Apoptosis	-	CRT HMGB1 HSP70	Upregulation of CD80, CD86, CCR5 and CCR7 on BC cells	DC activation	[30]
Chemoradiotherapy (Irradiation + Cisplatin) After anti-PD-1 treatment	Human UC + In vivo murine model and MB49 UC cell line	-	-	CRT HMGB1	Increased cytotoxic T cells	Objective response rate and overall survival	[31]
Capsaicin	T24 and SD48 human BC cell lines	Apoptosis	-	CRT ATP HSP70/90	-	Stimulate ICD	[32]
**n3**-polyunsaturated fatty acid docosahexaenoic acid	EJ Human BC cell lines	Apoptosis	-	CRT exposure	-	Stimulate ICD	[33]
**YB-1-selective adenovirus Xvir-N-31**	Multiple human BC cell lines and Orthotopic murine model	-	-	HMGB1 HSP70	-	Stimulate ICD	[34]
HPV non-replicant pseudovirions encoding for thymidine kinase (PsV-TK) in combination with Ganciclovir (GCV)	MB49 UC cell line and orthotopic murine model	Apoptosis	-	CRT HMGB1 IFN	Increased CD8+ T cells	Stimulate ICD	[35]
Norcantharidin (NCTD)	EJ and UMUC3 human BC cell lines, MB49 mouse cell lines and orthotopic murine model	Apoptosis	-	CRT	NCTD enhances autophagy, Increased CD4+ and CD8+ T cells	Promoted DC Maturation. NCTD reduced tumor growth. NCTD-induced ICD and increased survival	[36]
Recombinant adenovirus expressing CD40 ligand (Rad-CD40L) and 5-fluorouracil (5-FU)	MB49 UC cell line and Orthotopic murine model	Apoptosis	-	HMGB1 ATP	Rad-CD40L/5-FU combination treatment was more effective than each one alone	Stimulated ICD, Decreased tumor growth. Increased survival of the mice.	[37]
Hypericin-photodynamic (Hyp-PDT) or mitoxantrone (MTX)	AY27 cell lines and subcutaneous injection murine model	-	-	ATP HMGB1	Decreased ecto-CRT level	None of vaccinated rats showed tumor rejection.	[38]
Gemcitabine	T24 human BC cell line, G69 murine BC cell line and subcutaneous injection murine model	-	-	ATP CRT HMGB1 HSP70/90 ANXA1 PDIA3 IFN	CD8+ T cell response after PGE2 blockade	Gemcitabine vaccination did not affect tumor volume and survival. Induction of DAMPs by gemcitabine was not sufficient to induce ICD	[39]
Gemcitabine-cisplatin chemotherapy, Celecoxib and anti-PD1 antibody	G69 and G7 murine BC cell line and intraperitoneally injection murine model	-	-	CRT HSP70 HMGB1 IFN	CD8+ response increased after PGE2 blockade	Prostaglandin E2 blockade enhanced immunity and sensitized tumor to anti-PD1	[40]
Mitomycin C (MMC)	Multiple human UC cell lines, CT26 murine BC cell line and subcutaneous injection murine model	Apoptosis	-	HMGB1 CRT ATP	-	MMC induced ICD in short schedule treated cells. Cytoplasmic release of mitochondrial DNA, DC activation and induced ICD	[41]
Photodynamic therapy (PDT)	T24 human BC cell line, CT26 murine BC cell line and subcutaneous injection murine model	Apoptosis		CRT ATP HSP70/90	-	ROS-based ER stress induced ICD, DC maturation. Adaptive immune system activation	[42]
Newcastle Disease Virus (NDV), anti-PD-1 and anti CTLA4 monoclonal antibodies	Multiple human BC cell lines, MB49 UC cell line and flanks intradermally injection murine model	-	-	CRT IFN	-	Activation of innate immune pathway, induced ICD, increased immune infiltration plus delay of tumor growth and increased survival, upregulation of MHC I and II and PD-L1	[43]
Hypericin-based Photodynamic therapy (PDT)	T24 human BC cell line	-	-	CRT	Autophagy induced	Induced ICD, DC maturation,	[44]
Vesicular stomatitis virus containing the human GM-CSF transgene (VSVd51-hGM-CSF)	MB49 murine cell lines, 5637 and UM-UC-3 human BC cell lines, Huan BC tissue and orthotopic murine model	Necrosis	-	ATP CRT HMGB1 IFN	Enhanced immunogenic gene expression in MB49 cells	Immune cell activation, induced ICD, DC activation, reduced tumor volume and improved mice survival	[45]

**Table 2:** Overview of clinical trials involving ICD inducing agents.

ICD Inducer	Tumor Type	Type of Cell Death	Sample Size	DAMPs	Other Outcome	Immune Response	Side Effect	References
Coxsackievirus A21 (CVA21) + Mitomycin C (MMC)	Phase I trial, Patients with NMIBC	Apoptosis Necrosis	15	HMGB1 CRT IFN	Virally induced cytokines (IL6, IL1a, IL1b, IL23 and TNFα),	Upregulating IFN inducible genes, including both immune checkpoint inhibitory genes (PD-L1 and LAG3) and Th1-associated chemokines, as well as the induction of the innate activator RIG-I,	No grade 2 or higher side effects. Urinary tract infection responsive to antibiotic in 6/15 patients	[46]
Gemcitabine and Cisplatin (GC) plus Ipilimumab	Phase II trial, Patients with metastatic UC	-	36	No significant increase in serum HMGB1 levels were observed after treatment with two cycles of GC	No significant changes in immune cell subsets after GC alone. After the addition of ipilimumab, there was a significant expansion of peripheral blood CD4+ and a numerical increase in peripheral blood CD8+ cells	Improvement in survival associated with a post-ipilimumab expansion of peripheral blood CD4+ cells	Grade 3 or higher side effects in 81% of patients. Most common grade 3 side effects were hematologic. Immune related diarrhea in 11% of patients	[47]
Adenoviral vectors expressing CD40 ligand (AdCD40L)	Phase I/II trial, Patients with metastatic UC	-	8	IFN-γ	-	Reduced the load of malignant cells. Boosted immune activation	No adverse effects ascribed to the vector	[48]

## Appendix A. Search Strategies

**Medline (ovid), Central (ovid)**

((Antigen exposure).mp or (antigen presentation).mp or (immune system activation).mp or (immun* activation).mp or (immun* necrosis).mp or (immunogenic cell death).mp or ICD.mp) AND (exp Urinary Bladder Neoplasms or (Urinary Bladder Neoplasm*).mp or (bladder neoplasm*).mp or (bladder tumor*).mp or (bladder tumour*).mp or (bladder cancer*).mp or (Urinary Bladder cancer*).mp or (Urinary Bladder malignant tumor*).mp or Exp Carcinoma, Transitional Cell or Exp Ureteral Neoplasms or (upper tract urothelial cancer).mp).

**Embase**

((‘Antigen exposure’:ti,ab or ‘antigen presentation’:ti,ab or ‘immune system activation’:ti,ab or ‘immun* activation’:ti,ab or ‘immun* necrosis’:ti,ab or ‘immunogenic cell death’:ti,ab or ICD:ti,ab) AND (‘bladder tumor’/exp or ‘Urinary Bladder Neoplasm*’:ti,ab or ‘bladder neoplasm*’:ti,ab or ‘bladder tumor*’:ti,ab or ‘bladder tumour*’:ti,ab or ‘bladder cancer*’:ti,ab or ‘Urinary Bladder cancer*’:ti,ab or ‘Urinary Bladder malignant tumor*’:ti,ab or ‘transitional cell carcinoma’/exp or ‘ureter tumor’/exp or ‘upper tract urothelial cancer’:ti,ab)) AND [embase]/lim.
